# Development of iFOX‐hunting as a functional genomic tool and demonstration of its use to identify early senescence‐related genes in the polyploid *Brassica napus*

**DOI:** 10.1111/pbi.12799

**Published:** 2017-08-22

**Authors:** Juan Ling, Renjie Li, Chinedu Charles Nwafor, Junluo Cheng, Maoteng Li, Qing Xu, Jian Wu, Lu Gan, Qingyong Yang, Chao Liu, Ming Chen, Yongming Zhou, Edgar B. Cahoon, Chunyu Zhang

**Affiliations:** ^1^ National Research Centre of Rapeseed Engineering and Technology College of Plant Science and Technology Huazhong Agricultural University Wuhan China; ^2^ Department of Crop Science Benson Idahosa University Benin City Nigeria; ^3^ Department of Biotechnology College of Life Science and Technology Huazhong University of Science and Technology Wuhan China; ^4^ Jiangsu Provincial Key Laboratory of Crop Genetics and Physiology Yangzhou University Yangzhou China; ^5^ Center for Plant Science Innovation and Department of Biochemistry University of Nebraska‐Lincoln Lincoln NE USA

**Keywords:** iFOX‐Hunting system, functional genomic platform, *Brassica napus*, *Arabidopsis thaliana*, early leaf senescence, *BnACBP1‐like*, acyl‐CoA binding protein

## Abstract

Functional genomic studies of many polyploid crops, including rapeseed (*Brassica napus*), are constrained by limited tool sets. Here we report development of a gain‐of‐function platform, termed ‘iFOX (inducible Full‐length cDNA OvereXpressor gene)‐Hunting’, for inducible expression of *B. napus* seed cDNAs in Arabidopsis. A Gateway‐compatible plant gene expression vector containing a methoxyfenozide‐inducible constitutive promoter for transgene expression was developed. This vector was used for cloning of random cDNAs from developing *B. napus* seeds and subsequent Agrobacterium‐mediated transformation of *Arabidopsis*. The inducible promoter of this vector enabled identification of genes upon induction that are otherwise lethal when constitutively overexpressed and to control developmental timing of transgene expression. Evaluation of a subset of the resulting ~6000 *Arabidopsis* transformants revealed a high percentage of lines with full‐length *B. napus* transgene insertions. Upon induction, numerous iFOX lines with visible phenotypes were identified, including one that displayed early leaf senescence. Phenotypic analysis of this line (rsl‐1327) after methoxyfenozide induction indicated high degree of leaf chlorosis. The integrated *B. napus* cDNA was identified as a homolog of an *Arabidopsis* acyl‐CoA binding protein (ACBP) gene designated *BnACBP1‐like*. The early senescence phenotype conferred by *BnACBP1‐like* was confirmed by constitutive expression of this gene in *Arabidopsis* and *B. napus*. Use of the inducible promoter in the iFOX line coupled with RNA‐Seq analyses allowed mechanistic clues and a working model for the phenotype associated with *BnACBP1‐like* expression. Our results demonstrate the utility of iFOX‐Hunting as a tool for gene discovery and functional characterization of *Brassica napus* genome.

## Introduction

Reverse genetic studies based on loss‐of‐function and gain‐of‐function mutations are widely used to identify novel genes for understanding of basic processes in plants and for introduction of valuable variations in crop genomes (Kuromori *et al*., 2009). Usually, in the loss‐of‐function mutant system, RNAi (Wang *et al*., 2013), Ds‐transposon (G van Enckevort *et al*., 2005), T‐DNA insertional (Hirochika *et al*., 2004) or more recently CRISPR/Cas9 mutagenesis (Ma *et al*., 2015) are used to either knockout or disrupt gene activity. Indeed, many loss‐of‐function mutants have been generated for functional genomic studies in *Arabidopsis thaliana* (Alonso *et al*., 2003; Kuromori *et al*., 2004) and rice (G van Enckevort *et al*., 2005; Kolesnik *et al*., 2004). In contrast, the gain‐of‐function mutant systems have typically used, gene activation tags and FOX (full‐length cDNA overeXpressor gene)‐Hunting techniques to investigate gene function in plants. The activation tag method is based on the random insertion of transcriptional enhancers into the plant genome to induce ectopic, constitutive expression of genes adjacent to the insertion sites (Nakazawa *et al*., 2003; Weigel *et al*., 2000). A chemical‐inducible activation tagging system has been used to identify important genes controlling the vegetative‐to‐embryonic transition in Arabidopsis (Zuo *et al*., 2002)**.** FOX‐Hunting is based on overexpression of a single or limited numbers of full‐length random cDNAs from a target species or organ in individual transgenic plants (Ichikawa *et al*., 2006; Sakurai *et al*., 2011). The FOX‐Hunting approach has been effective for the identification of genes associated with a variety of traits including heat and salt tolerance and nitrogen metabolism (Albinsky *et al*., 2010; Yokotani *et al*., 2008, 2009a,b).

Reverse genetic approaches for functional genomic studies have been largely used for species such as *Arabidopsis* and rice that are more amenable to high‐throughput transformation. Unfortunately, functional genomic tools for identifying useful genes from many crop species, especially polyploids, are lacking. Our particular crop of interest is *B. napus* or rapeseed. Globally, rapeseed ranks third behind palm and soya bean as a source of vegetable oils and is the most important oilseed crop in the cooler climates of China, Canada and northern Europe (Hu *et al*., 2016). Rapeseed production faces significant challenges including significant losses globally to diseases such as clubroot and *Sclerotinia*‐induced stem rot (Rondanini *et al*., 2012). Yield enhancement and improvement of its seed oil content and composition are also major rapeseed breeding and biotechnological targets.

Currently, no loss‐of‐function or gain‐of‐function mutant collections are available for systematic analyses of rapeseed gene functions to support genetic improvement efforts. The lack of these tools for rapeseed is due in part to its large genome size (~1200 Mbp), multiple copy number of homologous genes and the low efficiency of existing transformation methods. In addition, the high frequency of gene redundancy resulting from its polyploid nature reduces the likelihood of obtaining phenotypes from loss‐of‐function mutations (e.g. T‐DNA insertions) in single genes. In addition, activation tagging for generation of gain‐of‐function mutants often results in activation of multiple genes distal from the T‐DNA or transposon insertion site, which complicates efforts to link specific genes to observed phenotypes (An *et al*., 2003; Sallaud *et al*., 2004). These difficulties associated with development and use of mutant populations in rapeseed are also encountered with many other polyploid crops including wheat.

FOX‐Hunting is one gain‐of‐function system that has potential utility for crops such as rapeseed that are less tractable for production of mutants for functional genomic studies. This method involves construction of a full‐length cDNA (fl‐cDNA) library from the species or organ of interest and cloning of the cDNAs under control of a constitutive promoter (e.g. CaMV35S) into a plant expression binary vector. The resulting library is then transformed into Arabidopsis for screening of ectopic expression phenotypes (Ichikawa *et al*., 2006; Nakamura *et al*., 2007). A limitation of the FOX‐hunting method is that genes that result in lethality or strongly reduced growth upon ectopic expression are missed in the FOX‐Hunting mutant screens (Papdi *et al*., 2008). In addition, strong constitutive promoters such as CaMV35S can cause transgene silencing (Du *et al*., 2008; Kei‐ichiro *et al*., 2005). To facilitate *B. napus* functional genomic studies and gene discovery, we have developed an inducible FOX‐Hunting system termed ‘iFOX‐Hunting’ involving the use of an inducible promoter for the expression of rapeseed seed cDNAs in *Arabidopsis*. In addition to the ability to identify genes that induce lethality or impaired growth, the use of an inducible promoter allows one to finely tune expression to specific developmental stages to gain more insights into gene function. This report describes the development of the iFOX‐Hunting system using a methoxyfenozide‐inducible promoter and its application especially to rapeseed functional genomic studies. As demonstrated, the close genetic relation of rapeseed and *Arabidopsis* allows for predictable translation of findings from the Arabidopsis screening system to rapeseed. We also show for the first time how the iFOX‐Hunting‐inducible promoter can be coupled with RNA‐Seq studies in rapeseed to gain basic insights into the functions of genes identified in mutant screens.

## Results

### Generation, evaluation and screening of Rapeseed iFOX mutant library

**Figure 1:**
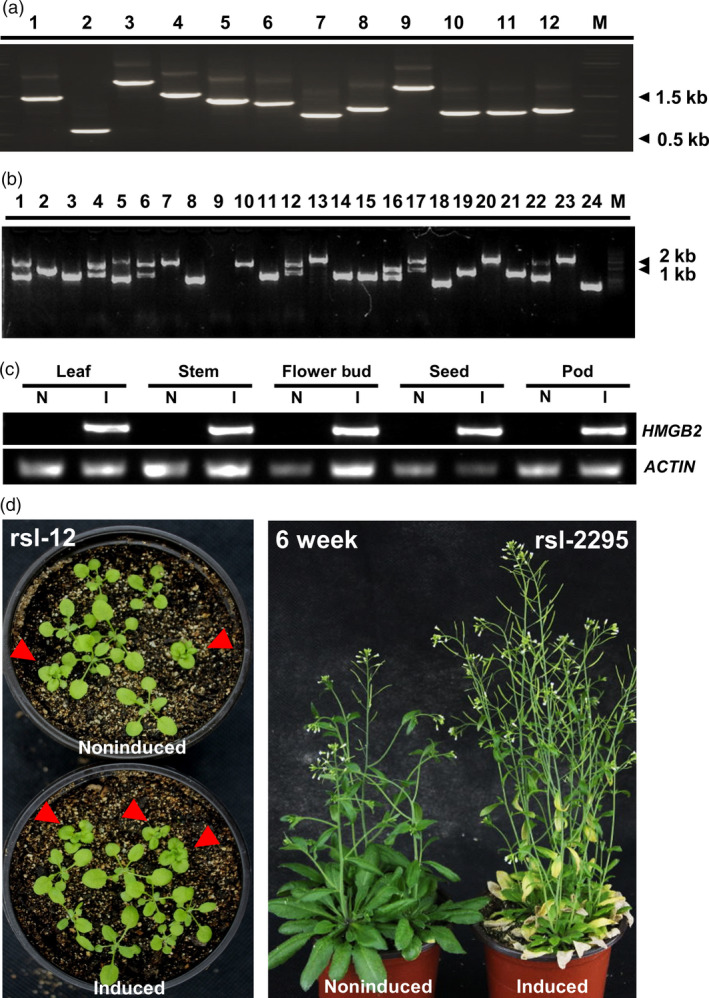
Evaluation of the full‐length cDNA transgenes in entry vector and in Arabidopsis. (a) and (b) Example of size distribution of the rapeseed cDNA in entry vector library and in Arabidopsis transformed lines. PCR‐amplified fragments including rapeseed cDNA were electrophoresed. Lane M, 2‐kb DNA size markers. (b) In lanes, 1, 4, 5, 6, 12, 16, 17 and 22 bands were amplified. (c) Transgene expression in different tissues‐induced and noninduced condition by RT‐PCR. Samples taken from 4 weeks mutant *rsl‐1375*, induced and noninduced after 36 h. Upper bands are the PCR fragments specific for *HMGB2*. Lower bands are Actin PCR fragments used for loading adjustment. (d) Example of a loss‐of‐function line *rsl‐12* and a gain‐of‐function line *rsl‐2295*. The phenotype of *rsl‐2295* line showing early senescence in induced condition. The phenotype of *rsl‐12* showing cabbage leaf phenotype in induced or noninduced condition, because of the separation in T2, the red arrows show the T–DNA insert mutant.

We took advantage of the Gateway^®^ cloning technology to establish a rapeseed full‐length cDNA (fl‐cDNA) entry vector. To eliminate deleterious effects associated with cDNA overexpression by a strong constitutive promoter, such as the CaMV35S promoter, we developed a conditional gene regulation vector, based on the interaction of a chemical inducer methoxyfenozide, that is compatible with the Gateway^®^ technology. This destination vector harbours methoxyfenozide‐responsive gene switch and a Basta resistance gene for selection of transformants. To evaluate the cDNA entry library, 10 000‐fold dilution of cDNA library was cultured overnight on LB solid medium and an average of 45 clones was counted, which could be interpreted as 1.35 × 10^7^ clones of the total library, sufficient to represent most of the genes expressed in *B. napus* seed. PCR insert fragment of randomly picked 347 clones ranged 0.5–2.0 kb with an average size at 1.26 kb (Figure 1a). Furthermore, a blast search of sequenced fragments revealed that 2% noncoding RNA, 27% non‐full‐length cDNA and 69% full‐length cDNA, which include 72% low abundant and 28% high abundant cDNAs, were contained in the entry library (Figure S1a and b). We used the Gateway LR Clonase II to catalyse the reaction of the entry vector with the destination vector containing the methoxyfenozide‐inducible expression system to generate the plant expression vector. The vector structure with restriction enzymatic sites is shown in Figure S1c. The plant expression library was introduced into Agrobacterium by electroporation and subsequently used for transforming Arabidopsis ecotype Columbia‐0 (Col‐0) by floral dip transformation (Clough and Bent, 1998).

Following floral dip transformation, we generated a total of >6000 T_1_ seed lines. Seed from transformed plants was geminated, and 4298 positive T_1_ transgenic plants were selected after screening with Basta. The size of fl‐cDNA inserts based on PCR analysis ranged from 1 to 2 kb (Figure 1b). Most of the fl‐cDNA (77%) amplified single cDNA fragments and had sequence size above 1.5 kb (Table S1). This inserted cDNA sequence size was comparable to that found in the library used for plant transformation (Figure S1a and b). Although copy number of transgenes was not surveyed in the recovered lines, it is assumed that one or more transgenes are likely present in the selected lines, as is typical for Agrobacterium‐based floral dip method in Arabidopsis. This ultimately necessitates additional functional confirmation of genes identified in the iFOX screen, as described in the example below.

### Observing visible phenotype of T_2_ generation in induced condition

**Figure 2:**
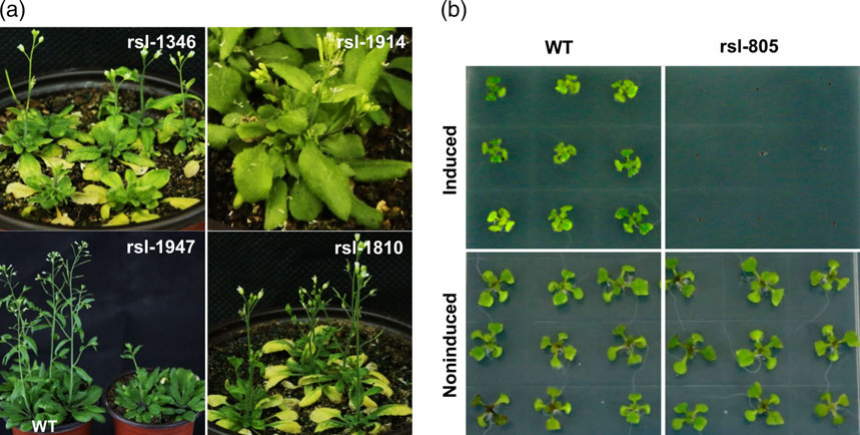
Phenotypes of the gain‐of‐function mutant lines. (a) The phenotype of the different mutant lines in induced condition. *rsl‐1346* and *rsl‐1810* show early leaf senescence phenotype, *rsl‐1947* show dwarf phenotype, and *rsl‐1914* show late flowering. (b) Comparison of mutant line *rsl‐805* in induced and noninduced MS medium for 12 days. The wild‐type seeds show normal germination and development in both condition, while *rsl‐805* did not germinated in induced condition. The concentration of the induced MS medium was 500 μL/L methoxyfenozide.

We subsequently established an efficient screening system to facilitate discovery of visible phenotypes resulting from inducible overexpression of rapeseed fl‐cDNAs in a selected portion of the T_1_ lines (1000 lines). First, we determined the optimal inducer concentration (61.3 μm) required to achieve maximal level of transgene transcript accumulation (Koo *et al*., 2004) (Figure S1e). Next, 200 positive T_1_ transgenic plants were self‐pollinated to generate T_2_ seed stock. The resulting T_2_ progenies were screened with methoxyfenozide, and visible phenotypic changes were identified in these transgenic lines under induced condition (Figure 1d). To determine whether the observed phenotype resulted from gain or loss of function, we planted the same lines from T_2_ seed stock under induced and noninduced conditions. Interestingly, no visible phenotypes were observed in most lines without induction. Meanwhile, phenotypic changes were observed in a number of the induced lines. Such mutations were considered to be gain‐of‐function mutations. However, a few transgenic plants displayed similar visible phenotypes under noninduced and induced conditions. Such lines were considered to be loss‐of‐function mutants due to insertional mutagenesis, although it cannot be excluded that the inducible promoter was ‘leaky’ in these lines. This process was repeated for over 3000 positive T_1_ plants advanced to the T_2_ generation. From this population, 37 transgenic candidates with visible morphological changes compared to noninduced lines and wild‐type plants were obtained. These included 31 gain‐of‐function mutants and 6 loss‐of‐function mutants (Figure S1d). The gain‐of‐function lines included 22 early leaf senescence mutants and two mutants displaying lethality (Table S2, Figure 2a and Data S1). These mutants maintained stable, inducible phenotypic expression through subsequent generations.

To determine whether transcript induction correlates with observed phenotypes following induction, we analysed the transgene expression pattern in different tissues of a randomly selected iFOX line (*rsl‐1375*) 36 h after induction. Enhanced transcript abundance was detected in all organs tested, which confirmed ectopic gene expression was achieved by inducible promoter (Figure 1c). We then selected 23 gain‐of‐function transgenic lines, rescued and sequenced their individual fl‐cDNAs. Sequence analysis revealed they had single fragment of rapeseed fl‐cDNA, and these fl‐cDNAs had *Arabidopsis* homologs coding for various protein with diverse functions (See Table 1 for more detail). Briefly, that is, Line *rsl‐1947* had dwarf phenotype and contained rapeseed fl‐cDNA with protein sequence predicted as At3G49910 homolog, a translation protein SH3‐like family involved in cold response. Induction of rapeseed fl‐cDNA integrated in Line *rsl‐1181* conferred narrow leave phenotype and its predicted sequence had no known function in Arabidopsis. Interestingly, Line *rsl‐805* showed a very strong lethal phenotype under induced condition (Figure 2b), and the recovered cDNA sequence had high protein similarity with Arabidopsis AT5G45890 annotated as *Senescence‐associated gene 12* (Table 1). This lethal phenotype of Line *rsl‐805* following induction confirmed earlier reports of deleterious effect of CAMV35S as a constitutive promoter for ectopic expression fl‐cDNA(s) (Papdi *et al*., 2008). This result also underscored the usefulness of the inducible promoter to identify genes deleterious genes and to controllably obtain phenotypes at desired developmental stages.

**Table 1:** Sequence analysis of selected 23 gain‐of‐function transgenic lines

Line	Homologous gene in Arabidopsis	Annotation in database	Query cover	Phenotype
rsl‐70	AT2G41280	Late embryogenesis abundant protein (M10)	73%	Early leaf senescence
rsl‐116	AT2G32930	Zinc finger nuclease 2	87%	Narrow leaves
rsl‐395	AT1G06760	Winged‐helix DNA‐binding transcription factor family protein	66%	Early leaf senescence
rsl‐486	AT3G43810	Calmodulin 7	100%	Early leaf senescence
rsl‐805	AT5G45890	Senescence‐associated gene 12	78%	Early leaf senescence/lethal
rsl‐935	AT3G02550	LOB domain‐containing protein 41	82%	Early leaf senescence
rsl‐1181	AT1G62870	Unknown protein	96%	Narrow leaves
rsl‐1300	AT1G51980	Insulinase (Peptidase family M16) protein	85%	Dwarf
rsl‐1327	AT5G53470	Acyl‐CoA binding protein 1	85%	Early leaf senescence
rsl‐1346	AT1G01720	NAC, ATAF	86%	Early leaf senescence
rsl‐1375	AT1G20693	High mobility group B2	87%	Early leaf senescence
rsl‐1436	AT5G62260	AT hook motif DNA‐binding family protein	77%	Early leaf senescence
rsl‐1472	AT1G04270	Cytosolic ribosomal protein S15	93%	Pale silique
rsl‐1479	AT1G33680	KH domain‐containing protein	70%	Early leaf senescence
rsl‐1512	AT5G56500	Cpn60 chaperonin family protein	84%	Yellow silique
rsl‐1752	AT4G25570	Ferric reductase transmembrane protein family	86%	Early leaf senescence
rsl‐1767	AT2G33880	Homeobox‐3	72%	Slow growth
rsl‐1810	AT5G19990	Regulatory particle triple‐A ATPase 6A	89%	Early leaf senescence/lethal
rsl‐1947	AT3G49910	Translation protein SH3‐like family protein	89%	Dwarf
rsl‐1988	AT1G75990	PAM domain (PCI/PINT associated module) protein	80%	Early leaf senescence
rsl‐2208	AT3G53970	Proteasome inhibitor‐related	74%	Early leaf senescence
rsl‐2295	AT5G47120	BAX inhibitor 1	88%	Bolting early
rsl‐2301	AT1G65720	Unknown protein	66%	Early leaf senescence

Sequencing the fragments in different lines and blasting them in TAIR to obtain the information of homologous genes in Arabidopsis.

### Characterization of early leaf senescence in rapeseed iFOX Arabidopsis line (*rsl‐1327*)

**Figure 3:**
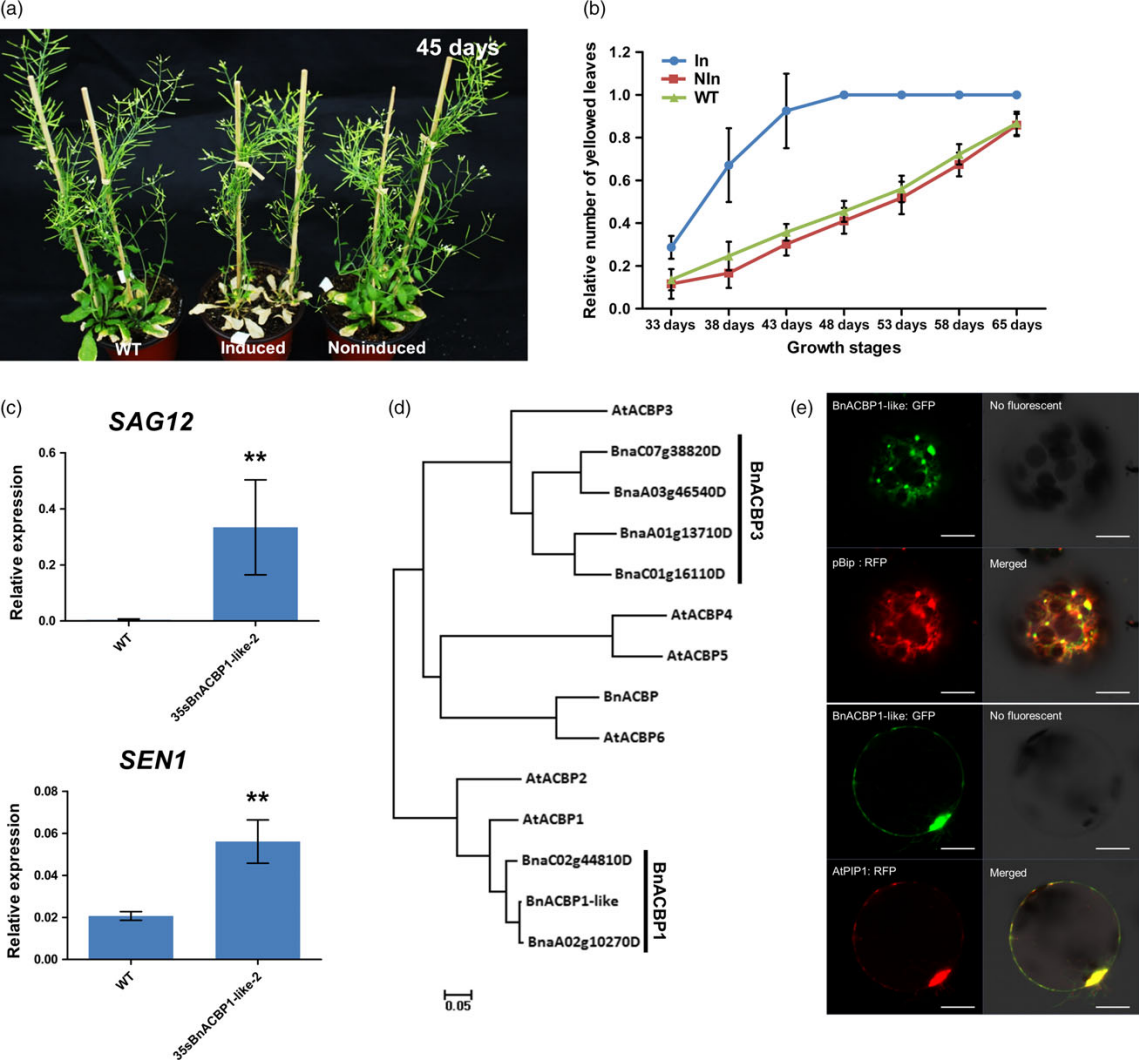
Characterization of early leaf senescence in iFOX Arabidopsis line (*rsl‐1327*). (a) The phenotype of *rsl‐1327* in induced condition. *Rsl‐1327* shows significantly early leaf senescence compared with WT and noninduced. (b) The relative leaf senescence ratio in different growth stage. Values are means ± (*n* = 96) of three independent experiments. (c) The transcript levels of senescence mark genes *SAG12*, *SEN1* in 4 weeks *35sBnACBP1‐like* seedling leaves. The values are the means ± SD for three biological replicates (**P < 0.01). (d) Evolutionary relationships of ACBP1 in Arabidopsis and *Brassica napus*. The analysis involved 13 amino acid sequences. Evolutionary analyses were conducted in MEGA7. (e) Subcellular localization of *BnACBP1‐like* in protoplast transformation. pBip‐RFP is an endoplasmic reticulum‐localized marker; AtPIP1, RFP is plasma membrane marker. At least three independent transformation experiments were performed using the two constructs.

Among the 22 lines that displayed early leaf senescence phenotype in the T_2_ generation, line *rsl‐1327* showed dramatic leaf senescence just after bolting under induced condition (Figure 3a) and was selected for further analysis. Morphological characters such as plant height, leave shape, floral organ formation, flowering time and fruit set were not significantly different between induced and noninduced plants (Control) (Table S3). However, leaf ageing rate and the number of senesced leave (Figure 3b) varied significantly. Consistent with programmed cell death (PCD) (Lim *et al*., 2007), the chlorophyll content of *rsl‐1327* was significantly lower than that of the control (Figure S2a), and the relative expression of two senescence‐related marker genes (*SAG12, SEN1*) was significantly up‐regulated in *rls‐1327* plants compared to wild‐type plants (Figure 3c).

To identify the transgene integrated in the *rsl‐1327* line, we amplified the fl‐cDNA and sequenced it with insert specific primers. Phylogenetic analysis revealed *Rsl‐1327* homolog gene in *Arabidopsis* is *ACBP1* (AT5G53470) and acyl‐CoA binding protein (Xiao and Chye, 2011), while in *B. napus* it is similar to *BnACBP1* (BnaA02g10270D) and NCBI BLAST^®^ indicated that the *Rsl‐1327* protein sequence is BnACBP1‐like (Figure 3d). Results from subsequent studies using GFP‐tagged protein expressed in protoplasts were consistent with plasma membrane and ER‐localization of the BnACBP1‐like protein (Figure 3e).

**Figure 4:**
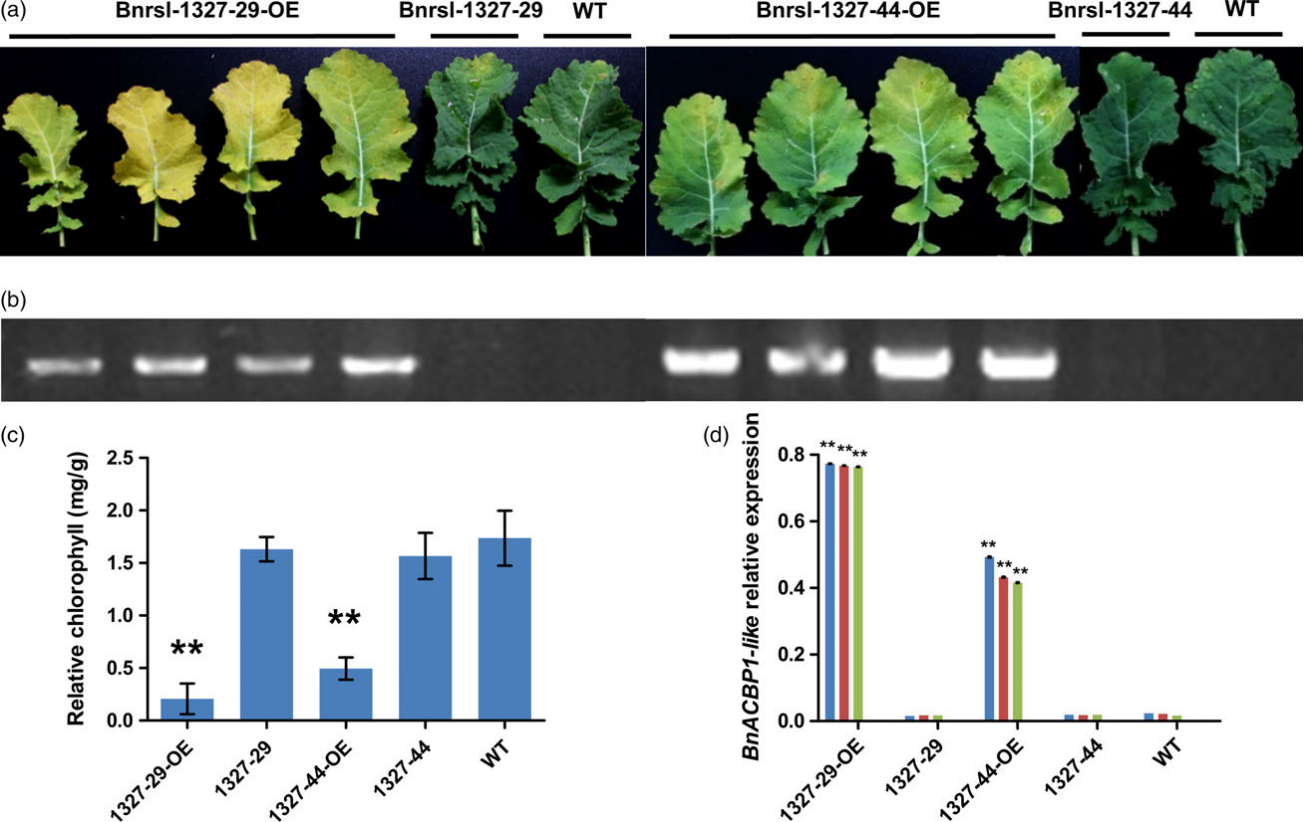
The characteristic of two different *BnACBP1‐like* overexpression transformation lines in *B. napus*. (a) The phenotype of *BnACBP1‐like* overexpression transformation lines. After 8 days dark treatment, the leaves of two lines show different senescence degree, the T‐DNA insert plants all show senescence, no insert and wild type did not show significant senescence. All the leaves come from 3 months seedling at the same position. (b) PCR analysis of the T‐DNA insert in two Brassica lines. Insert specific primers were used. (c) The *BnACBP1‐like* relative expression in different lines by qRT‐PCR. RNA was extracted from 3‐month leaves. Error bars indicate s.d. from three technical replicates. The transcript levels of each gene were normalized to Actin7. (d) Relative chlorophyll contents of two different *BnACBP1‐like* overexpression transformation lines. After 8 days of dark treatment, total chlorophyll content was measured and normalized per gram fresh weight of sample. Asterisks indicate significant difference from the wild type at the same treatment (*P < 0.05 or **P < 0.01). Values are means ± SD (*n* = 3) of three independent experiments.

To confirm that induced‐overexpression of the *BnACBP1‐like* triggered the accelerated early leaf senescence phenotype, we performed independent retransformation of Arabidopsis wild‐type Col‐0 lines with the recovered fl‐cDNA fused to a constitutive promoter (CaMV35S). After retransformation, the early senescence phenotype was observed in all regenerated plants (Figure S2b and c). The chlorophyll content of these plants was also significantly reduced compared to wild‐type plants (Figure S2d). Similar phenotypes were also observed with transformation of the *BnACBP1‐like* cDNA under control of the CaMV35S promoter in *B. napus* (Figure 4a). In the two confirmed transformation events (Figure 4b). When premature leaf senescence was assessed in detached leaves, leaves from two different transgene lines showed accelerated senescence marked by yellowing 8 d after detachment, while those of the wild type remained green (Figure 4a). In particular, lines *Bnrsl‐1327‐29‐OE* and *Bnrsl‐1327‐44‐OE* plants had very severe leaf etiolation and significantly low chlorophyll content after 2 months (Figure 4c). Consistent with these phenotypes, the *BnACBP1‐like* transcript level in *Bnrs1‐1327‐OE* lines was approximately 80‐fold higher than in the control lines (Figure 4d). Together, these results demonstrate that the *BnACBP1‐like* overexpression phenotype in *B. napus* is essentially the same as observed in the Arabidopsis *rsl‐1327* line.

### Transcriptome analysis of *rsl‐1327* lines

To gain insights into the function of *BnACBP1‐like*, RNA‐Seq studies were conducted using four‐week‐old *rsl‐1327* plants. Samples were collected from noninduced and induced plants at 2 h and 4 h after application of water (noninduced) or methoxyfenozide (induced) (Figure S3a). The results of RNA‐Seq data preprocessing and read alignment are reported in Table S4, Data S1 (including Figures S3b‐d, S4d and S5). At 2 h, significant changes (*P* < 0.01, FDR = log2FC > 1) in gene expression were detected in 608 genes between the induced and noninduced plants (Table S5). Approximately 87% of these genes were up‐regulated and 13% were down‐regulated in the induced versus noninduced plants (Table S6). Functional annotation revealed that 575 genes were assigned to cellular component, 535 genes were assigned to molecular functions, and 558 genes were assigned to biological process (Figure S4a‐c). The result of gene enrichment test revealed significantly (FDR < 0.05, *P* < 0.01) enriched genes mostly related to defence responses, which included genes related to jasmonic acid (JA) biosynthesis (AT1G76680, AT2G06050, AT3G25760, AT3G25780, AT1G72520); signalling processes (AT1G19180, AT5G47220, AT3G11820); and JA stimulus (AT1G18570, AT2G34810, AT5G13220); and oxylipin biosynthetic and metabolic process (AT2G06050, AT3G25760, AT1G17420, AT2G26560, AT1G72520). Others up‐regulated genes encoded SNARE superfamily proteins (AT3G11820, AT3G52400, AT4G23210) and transmembrane receptor genes (AT5G41750, AT5G44910, AT2G39200, AT1G66090, AT5G41740, AT1G51270, AT1G29690) that function in signal transduction, immune system response and PCD (Table S7).

**Figure 5:**
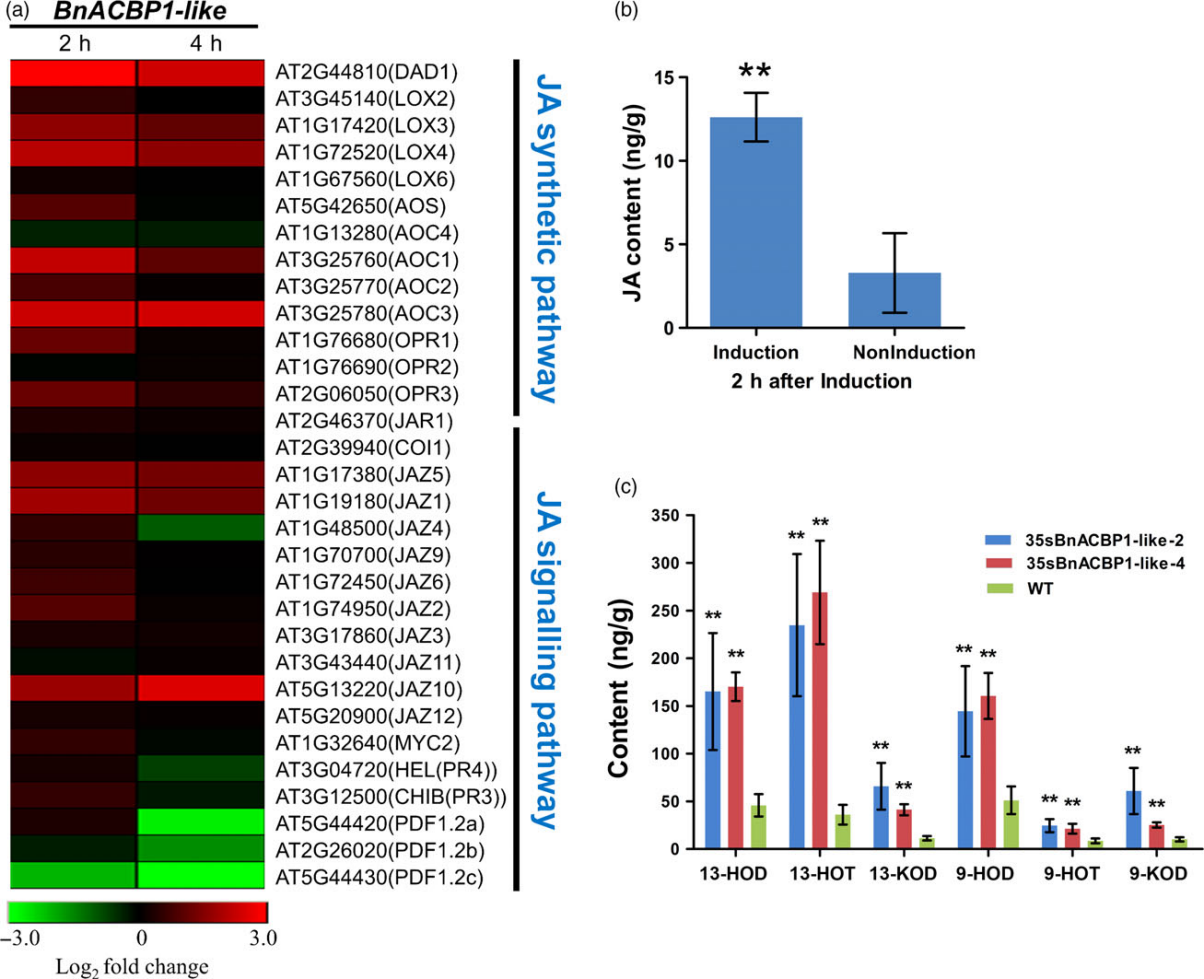
RNA‐seq results and JA and oxylipin content in response to induction. (a) The heat map of JA synthetic and signalling pathway after induced 2 h and 4 h. (b) The JA levels were enhanced after 2‐h induction in three‐week‐old seedlings. The values are the means ± SD for six biological replicates. The asterisks indicate statistically significant differences between the transgenic and WT plants (*P < 0.05, **P < 0.01). (c) Major oxylipin compositions for 9 of 13 hydroxy‐FAs and keto‐FAs in the WT and *35sBnACBP1‐like* transgenic line leaves. 9‐ / 13‐HOT, 9‐ / 13‐hydroxy octadecatrienoic acid; 9‐ / 13‐HOD, 9‐ / 13‐hydroxy octadecadienoic acid; 9‐ / 13‐KOD, 9‐ / 13‐keto octadecadienoic acid. The values are the means ±s.d. for three biological replicates. The asterisks indicate statistically significant differences between the transgenic and WT plants (*P < 0.05, **P < 0.01).

After 4 h of induction, fewer responsive genes (123) based on *P* < 0.01, *FDR* = log2FC > 1 were observed (Table S8). About 89% of these differentially expressed genes (DEG) showed significant up‐regulation, while 11% were down‐regulated (Table S6). Functional annotation and enrichment (FDR < 0.05, *P* < 0.01) uncovered significant biological processes similar to what has been observed in the 2 h treatment, including enrichment of genes related to JA stimulus and oxylipin metabolic process, oxidative stress and defence responses, and PCD (Table S9). Furthermore, evaluation of expression profile revealed significant co‐expression relationship between genes involved JA biosynthetic and signalling pathways (Figure 5a). In this regard, JA was of interest and prompted further analysis.

### Inducible expression of *BnACBP1‐like* conferred senescence phenotype of *rsl‐1327* by promoting high accumulation of JA and oxylipins

To confirm whether the significant up‐regulation of JA synthesis and signalling genes resulted from inducible expression of *BnACBP1‐like*, we measured JA content in both induced and control plants 2 h after inducer treatment. Consistent with the RNA‐Seq results, which showed significant up‐regulation of JA genes in early response to *BnACBP1‐like* inducible expression, JA content was significantly high in induced lines compared to water‐treated control plants (Figure 5b). This finding suggests that JA may play a significant role in promoting accelerated leaf senescence phenotype of the iFOX line *rsl‐1327* (He *et al*., 2002).

Given the overlap in the enrichment of genes related to JA, oxylipin and PCD and reports that JA enhances expression of several oxylipin genes through a feedback loop that amplifies signal transduction and cell death (Savchenko *et al*., 2014; Sun *et al*., 2014), we measured the oxylipin levels in Arabidopsis lines constitutively expressing *BnACBP1‐like* in comparison with wild‐type Col‐0. The results shown in Figure 5c revealed very high accumulation of oxylipins: 9‐HOD (9‐hydroxy linoleic acid), 9‐HOT (9‐hydroxy linolenic acid), 13‐HOD (13‐hydroxy linoleic acid), 13‐HOT (13‐hydroxy linolenic acid) and 9‐KOD (9‐keto linoleic acid), 13‐KOD (13‐keto linoleic acid) in the transgenic lines, confirming that ectopic expression of *BnACBP1‐like* induced metabolism of oxylipins. Together, these results suggest that *BnACBP1‐like* could mediate early leaf senescence through induction of JA and oxylipin signal transduction. Moreover, as JA is a signalling molecule, any significant changes in its level of biological activity will constitute an indirect regulation of metabolic, developmental and defensive processes in *rsl‐1327* (Devoto and Turner, 2003). Additionally, because the sublocation of *BnACBP1‐like* protein is in the endoplasmic reticulum and plasma membrane (Figure 3e), it is tempting to speculate that *BnACBP1‐like* might indirectly induce senescence‐associated PCD through unsaturated fatty acid metabolism via a pathway that involves lipoxygenase genes.

## Discussion

The identification of *BnACBP‐1like* in the studies reported here highlights advantages of the iFOX‐Hunting as a functional genomic tool, particularly compared to the previously described FOX‐Hunting system. Because its expression induces an early senescence phenotype, *BnACBP1‐like*, for example, may not have been identified without the use of an inducible promoter. In addition, the ability to translate findings from ectopic expression in Arabidopsis to rapeseed was shown by the observation of a similar early senescence phenotype from constitutive expression of *BnACBP1‐like* in transgenic *B. napus* lines. Furthermore, the inducible promoter in the iFOX‐Hunting system was particularly advantageous for gene function characterization by unravelling the network of genes responsive to *BnACBP1‐like* expression through the combined use of transcriptomic analyses and metabolomics. With a noninducible promoter, it is likely that this network of genes and the temporal aspects of the network's response to *BnACBP1‐like* expression would not be so clearly defined.

We produced about 6000 transgenic lines using the iFOX‐Hunting system. From this population, 4298 positive T_1_ lines were obtained that harboured the inducible plant expression vector and the rapeseed fl‐cDNAs. Size distribution pattern and sequence analysis of fl‐cDNAs in randomly selected iFOX mutants were not significantly different from fl‐cDNA contained in the entry vector (Figure S1a and b). Although the rapeseed seed‐specific fl‐cDNAs used in the study were not normalized, it is interesting that the diversity of fl‐cDNAs was largely maintained in the iFOX lines (Figure S1a and b). Future application of the iFOX strategy would likely benefit from normalization to increase the diversity of the genes screened.

From the 4298 confirmed transgenic lines, 37 T_2_ lines with altered visible phenotypes were identified using the methoxyfenozide inducer. Line *rsl‐1327* was chosen for gene characterization as an example of the application of iFOX‐Hunting in functional genomic research. This line was found to contain a cDNA corresponding to the *BnACBP1‐like* gene (BnaA02g10270D), which shares the highest identity to the Arabidopsis *ACBP1* gene (AT5G53470) (Figure 3d). In contrast to our findings with the *BnACBP1‐like* gene, it was previously reported that overexpression of *ACBP1* in Arabidopsis does not induce an early senescence phenotype (Lung and Chye, 2016). Instead, a phenotype similar to that observed with overexpression of the *BnACBP1‐like* gene in Arabidopsis and rapeseed was reported for overexpression of the more distantly related Arabidopsis *ACBP3* gene (Xiao *et al*., 2010). These findings indicate that although our results with *BnACBP1‐like* gene expression are similar in Arabidopsis and rapeseed, it is not possible to predict the functions of rapeseed genes based solely on high levels of identity with Arabidopsis genes.

### *BnACBP1‐like* mediates early leaf senescence in *rsl‐1327* through apparent induction of senescence‐associated PCD

**Figure 6:**
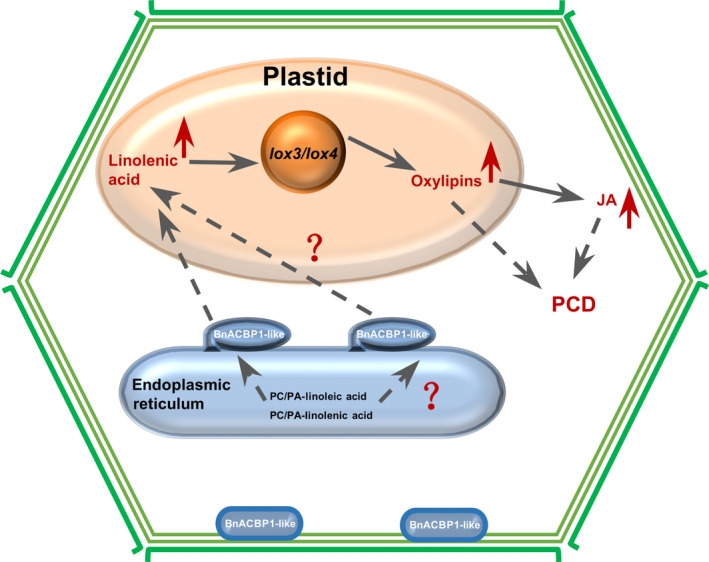
Proposed model defining the role of *BnACBP1‐like* in leaf early senescence. BnACBP1‐like may interact with PA/PC of linoleic acid and linolenic acid in ER. Recombinant AtACBP1 has been previously shown to bind PA and PC (Du *et al*., 2013). Possibly, BnACBP1‐like enhances PC/PA exchange and increases the linolenic acid content in plastids. The resulting substrate increase, in turn, leads to oxylipin accumulation by LOX 3/4 catalytic oxidation and/or JA accumulation, leading to PCD induction.

Our findings with *BnACP1‐like* are consistent with a role of this gene in PCD induction. These findings include the severe leaf chlorosis of *rsl‐1327* plants after induction, up‐regulation of senescence marker genes, and increased JA and oxylipin levels (9‐HOD/T, 13‐HOD/T and 9/13‐KOD) (Sun *et al*., 2014). Notably, it is well documented that jasmonates initiate adaptive defence processes that lead to senescence, and oxylipins mediate ROS (reactive oxygen species) production and cell death in plants (Fan *et al*., 2013; Montillet *et al*., 2005). Specifically, in our RNA‐Seq data, we observed significant increase in the expression levels of lipoxygenase 3 (AT1G17420 = log2FC: 1.61711) and lipoxygenase 4 (ATIG72520 = log2FC: 2.09071), genes known to be involved in fatty acid hydroperoxide formation. Lipoxygenases catalyse the oxygenation of fatty acids by addition of molecular oxygen to unsaturated fatty acids to yield an unsaturated fatty acid hydroperoxide (Schneider *et al*., 2007). Additionally, these LOX‐hydroperoxy fatty acids serve as precursor of diverse oxygenated fatty acids including jasmonates in plant (Caldelari *et al*., 2011; He *et al*., 2002), and studies have reported that LOX‐dependent hydroperoxide fatty acid formation is required for hypersensitive cell death development in plant, such as cotton (Jalloul *et al*., 2002; Marmey *et al*., 2007), pepper (Hwang and Hwang, 2010) and tobacco (Garcia‐Marcos *et al*., 2013). For these reasons, we hypothesize that BnACBP1‐like may interact with linoleic acid and linolenic acid in PC/PA of the ER. Recombinant AtACBP1 has been previously shown to bind PA and PC (Du *et al*., 2013). Possibly, BnACBP1‐like enhances PC/PA exchange and enhances the linolenic acid content in plastid. The substrate increase might lead to oxylipins accumulation by LOX 3/4 catalytic peroxidation, and this may directly cause PCD or indirectly cause JA levels to increase and then lead to senescence‐associated PCD. Based on the above hypothesis, we proposed a potential working model for role of *BnACBP1‐like* in leaves early senescence upon inducible overexpression (Figure 6). This model shows major cellular and physiological interaction in response to inducible overexpression of *BnACBP1‐like* in the iFOX line *rsl‐1327*. Given that the fl‐cDNA library screened by iFOX‐Hunting was prepared from developing seeds, it is likely that *BnACBP1‐like* has functions related to seed metabolism. Although this gene may also mediate fatty acid oxygenation via lipoxygenases in seeds, additional studies are needed to confirm this.

Although the iFOX‐Hunting system was used for visual screening of Arabidopsis phenotypes associated with the expression of a seed library prepared from rapeseed, this system can be applied to a wide range of screens for identification of gene function especially for polyploids crops species. For example, we have conducted additional screens, including gas chromatography (GC) of fatty acid methyl esters of seed oils from induced plants, to find new gene functions in rapeseed, including those related to oil metabolism.

The iFOX‐Hunting methodology can be easily adapted to the high‐throughput characterization of genes from other plant species as well as genes from specific organs, developmental stages or conditions (e.g. water stress). The example reported here also highlights the value of the inducible promoter of the iFOX‐Hunting system to identify genes whose ectopic expression is deleterious to growth and for use in transcriptomic studies to further dissect the function of genes identified from screens of the mutant populations. Overall, the iFOX‐Hunting method described here is a tool to expand plant functional genomic studies that is especially useful for crops such as rapeseed that currently lack high‐throughput transformation protocols and have considerable gene redundancy arising from polyploidy.

## Experimental procedures

### Plant materials and growth conditions

Arabidopsis plants (Wide‐type Col‐0) and the transformed lines were grown at 22 °C under a photoperiod conditions (16‐h light and 8‐h dark).

### Construction of *B. napus* fl‐cDNA expression library, plant transformation and selection

A rapeseed fl‐cDNA iFOX expression vector was produced according to the method described by Weiste *et al*. (2007) with slight modification. Briefly*,* seeds at different development stages were collected. Total RNA was isolated by CTAB, the mRNA was purified from total RNA using illstra™ mRNA Purification Kit, and equal amount of mRNA were pooled to obtain composite sample for cDNA library construction. Using the Gateway® cloning technology, first we established an enriched rapeseed full‐length cDNA entry vector (Weiste *et al*., 2007). The M13 primers were used to test and evaluate the cloned insert. Next, a Gateway® compatible destination 24101‐1 vector was constructed to include methoxyfenozide‐chemical‐inducible promoter (Koo *et al*., 2004) and the resistant gene for Basta. Following site‐specific recombination, the entry vector containing the rapeseed fl‐cDNA was transferred to the destination vector. The resulting binary vector was subsequently transformed into *Escherichia coli*. Afterwards, plasmid mixture extracted from transformed *Escherichia coli* containing the destination vectors was used to transform *Agrobacterium tumefaciens* GV3101. The resultant transformants harbouring the destination vector were selected by spectinomycin. A pool of these selected Agrobacteria was grown in liquid medium for plant transformation.

Arabidopsis plants were transformed by the floral dipping method using Agrobacterium (GV3101). Subsequently, leaves randomly selected from T_1_ plants were used for DNA extraction and PCR analysis to confirm fl‐cDNA diversity. Transformed T_1_ seeds were selected with 120 mg/L of Basta solution, and T_2_ seeds were screened for visible phenotype with 100 μL/L methoxyfenozide (Dow AgroSciences LLC 22.6%w/v). Phenotypes were scored based on morphological changes such as germination, leave size, shape and colour. Other parameters include flowering time and senescence. All plants showing visible phenotypes were transferred to a new growing tray. Rosette leaves were collected from T_2_ plants showing visible phenotype for further analysis.

### Genomic DNA isolation, PCR and sequencing

To identify integrated cDNAs, genomic DNA prepared from leaves of randomly selected 1064 T_1_ transgenic plants was the template for PCR amplification with primers complementary to vector sequences flanking the attB1 and attB2 sites (RS‐LBRY‐L985: GAGGACACGCTGAACGATGAGGACACGCTGAACGAT and Plinex‐r: CTGGTGATTTTTGCGGACTCCTGGTGATTTTTGCGGACTC). The PCR condition was 95 °C for 30 s for denaturation, 55 °C for 30 s for annealing and 72 °C for 60 s for elongation. The PCR products were gel purified and sequenced with the same primers. The identity of the transcript was revealed by sequence homology search using the TAIR BLAST tool. To validate the phenotype conferred by the inducible expression of fl‐cDNA, the cDNA was isolated and inserted into pGly35sRed3 expression vector driven by 35S promoter for Agrobacterium‐mediated transformation of Arabidopsis wild‐type Col‐0.

### RNA extraction and RT‐PCR

To evaluate the expression pattern of transgenes in different plant tissues after induction, semi‐quantitative RT‐PCR was performed on randomly selected mutant *rsl‐1375*. The TRIzol Reagent Kit (Ambion™) was used following manufacturer's instructions to extract total RNA from leaf, stem, flower bud and pod, respectively, of 4‐week‐old plant. First‐strand cDNAs were synthesized from each RNA preparation using the Thermo Scientific RevertAid Kit following manufacturer's instructions. The specific sequences of each of the primer pairs used in semi‐quantitative reverse transcription (RT‐PCR) are listed in: 1375‐f TGCTAAAGCAGCAGGTCGCA; 1375‐r ACACAGACTTGTCAGATTCC.

### Subcellular localization

PCR‐generated open reading frame of *BnACBP1‐like* without stop codon was subcloned in‐frame upstream of the GFP gene in the 35S‐GFP vector (Bottanelli *et al*., 2012). The construct was validated by sequencing (Forward primer: GACCGGTCCCGGGGGATCCATGGGTGTTGATTGGTTT, reverse primer: CCTTGCTCACCATGGATCCATCAGAATCCTTCTTCTCTC). Next tobacco (*Nicotiana benthamiana*) leaf protoplasts were isolated according to Aggarwal *et al*. (2014). The resulting constructs were transiently expressed in tobacco protoplast according to the method described by Batoko *et al*. (2000). Tobacco leaf protoplasts were isolated according to Aggarwal *et al*. (2014), subsequently, GFP signal was detected at room temperature after 24 h of expression with confocal fluorescence microscopy (Zeiss, LSM510 Meta, Carl Zeiss Germany).

### RNA sequencing

For RNA‐Seq, total RNA was extracted according to The TRIzol Reagent Kit RNA quality and quantity were determined using a Nanodrop 8000 (Thermo Scientific, Wilmington, DE) and a Bioanalyzer 2100 (Agilent, Santa Clara, CA). Before RNA extraction, *rsl‐1327* transgenic line was sprayed with the inducer ‘methoxyfenozide’, for control, and *rsl‐1327* transgenic lines were water sprayed. Leaves were collected after 2, 3, 4 and 5 h of inducer and control treatment. All samples were collected in three biological replicates. Afterwards, samples from each biological replicate at each time point were pooled. For RNA sequencing, only sample from 2 h and 4 h were used. In total, 12 samples were used to construct cDNA library with Illumina^®^ TruSeq™ RNA Sample Preparation Kit following the manufacturer's instructions. All samples were sequenced using an Illumina HiSeq 2000 sequencer at the National Key Laboratory of Crop Genetic Improvement, Huazhong Agricultural University.

### Analysis of sequence data

After image analysis, estimation of error and base calling with Illumina Pipeline, a hundred‐bp paired‐end sequences data were generated (paired‐end reads that were 100 bp in length). Next, reads from different samples were identified in the sequence data using indexed primers and low‐quality reads were removed using NGS QC tool kit as described by Wu *et al*. (2016). Reads that passed the QC were considered suitable for further analysis after passing quality control.

Afterwards, short‐read alignment and mapping of reads were carried on Arabidopsis genome annotation (TAIR 10) with the software TopHat v2.0.11 using the default parameters (Trapnell *et al*., 2009). Only, uniquely mapped reads were considered for gene expression analysis. Cufflinks v2.2.1 programme (Trapnell *et al*., 2010) was used to estimate transcript abundance and differential gene expression. Reads mapping to annotated transcripts were summed for each gene model and normalized by FPKM. Differentially expressed genes (DEGs) were identified with Cuffdiff implemented in Cufflinks software, and FDR was set at *P* value < 0.01, log2FC = >1.

For functional annotation, enrichment analysis, cluster and pathway analysis differentially expressed genes showing significant enrichment in response to control and induced treatments and comparisons were analysed using TAIR (http://www.arabidopsis.org/tools/bulk/go/index.jsp), Agrigo (Du *et al*., 2010), Genesis (Sturn *et al*., 2002) and KEGG databases.

### Validation of RNA‐Seq data

Quantitative real‐time PCR (qRT‐PCR) was performed on cDNA obtained from one of the biological replicates belonging to control and induced treatment, which was used for RNA sequencing. First‐strand cDNA was synthesized using Thermo Scientific RevertAid Kit following manufacturer's instructions. qRT‐PCRs were performed with SYBR Green Premix system (Newbio Industry) and specific primers (Data S1 and Table S10) using the CFX Connect™ Real‐Time PCR Detection System (BIO‐RAD, Hercules, CA). The expression profiles of 22 genes were analysed, with Actin7 (AT5G09810) used as constitutive gene for normalization. PCR conditions were 95 °C for 1 min, followed by 44 cycles at 95 °C, 12 s, 60 °C, 30 s and 72 °C, 30 s. After cycling, melting curves of the reaction were run from 55 °C to 95 °C. Relative expression was calculated with the software LINREG, as described by Ramakers *et al*. (2003).

### Measurement of jasmonic acid and oxylipin content

Quantification of JA and oxylipin was performed as described by Sun *et al*. (2014). Samples for JA and oxylipins analysis were prepared in parallel, six replicates for each according to Liu *et al*. (2012). For JA, 10 ng (±) – 9, 10 – dihydro – JA (Sigma) was added to each sample as internal standard. The samples were stored at −80 °C before the quantification. The JA levels were quantified using an HPLC‐MS/MS system (AB SCIEX Triple Quad 5500 LC/MS/MS) with JA (Sigma) as the external standards. To quantify oxylipin levels, we used 9‐ / 13‐HPOD, 9‐ / 13‐HPOT, 9‐ / 13‐HOD, 9‐ or 13‐HOT or 9‐ / 13‐KOD (Cayman Chemical Co) as the external standard.

## Supporting information


**Figure S1** Evaluation of the mutant library and Arabidopsis iFOX line (rsl‐1327).

**Figure S2** Characterization of *35sBnACBP1‐like* and the chlorophyll content of *rsl‐1327* in different growth stage.

**Figure S3** Experimental design of RNA‐Seq and data quality assessment.

**Figure S4** GO annotation and cluster analysis of RNA‐Seq data.

**Figure S5** qRT‐PCR validation of RNA‐Seq data.

**Table S1** Summary of PCR analysis of transgenes.

**Table S2** Number of gain‐of‐function and loss‐of‐function mutants.

**Table S3** Growth stages for induced and non‐induced (control) mutants.

**Table S4** Summary of RNA‐Seq read alignment results.

**Table S5** List of all differentially expressed genes in the pairwise comparison of non‐induced and induced at 2 h time point.

**Table S6** Evaluation of significant up‐ and down‐regulated genes in each pairwise comparison between time points.

**Table S7** Functional enrichment analysis of DEGs at 2 h timepoint.

**Table S8** List of all differentially expressed genes in the pairwise comparison of non‐induced and induced at 4 h time point.

**Table S9** Functional enrichment analysis of DEGs at 4 h timepoint.

**Table S10** Summary of quantitative real‐time PCR (qRT‐PCR) primer.

**Data S1** Loss of function phenotype and RNA‐Seq data analysis.

Supplementary File
