# Supplementary figures and images for: Development of iFOX‐hunting as a functional genomic tool and demonstration of its use to identify early senescence‐related genes in the polyploid Brassica napus
Source: Plant Biotechnol J. 2017 Aug 22;16(2):591–602. doi: 10.1111/pbi.12799 (PMC5787830; doi:10.1111/pbi.12799)

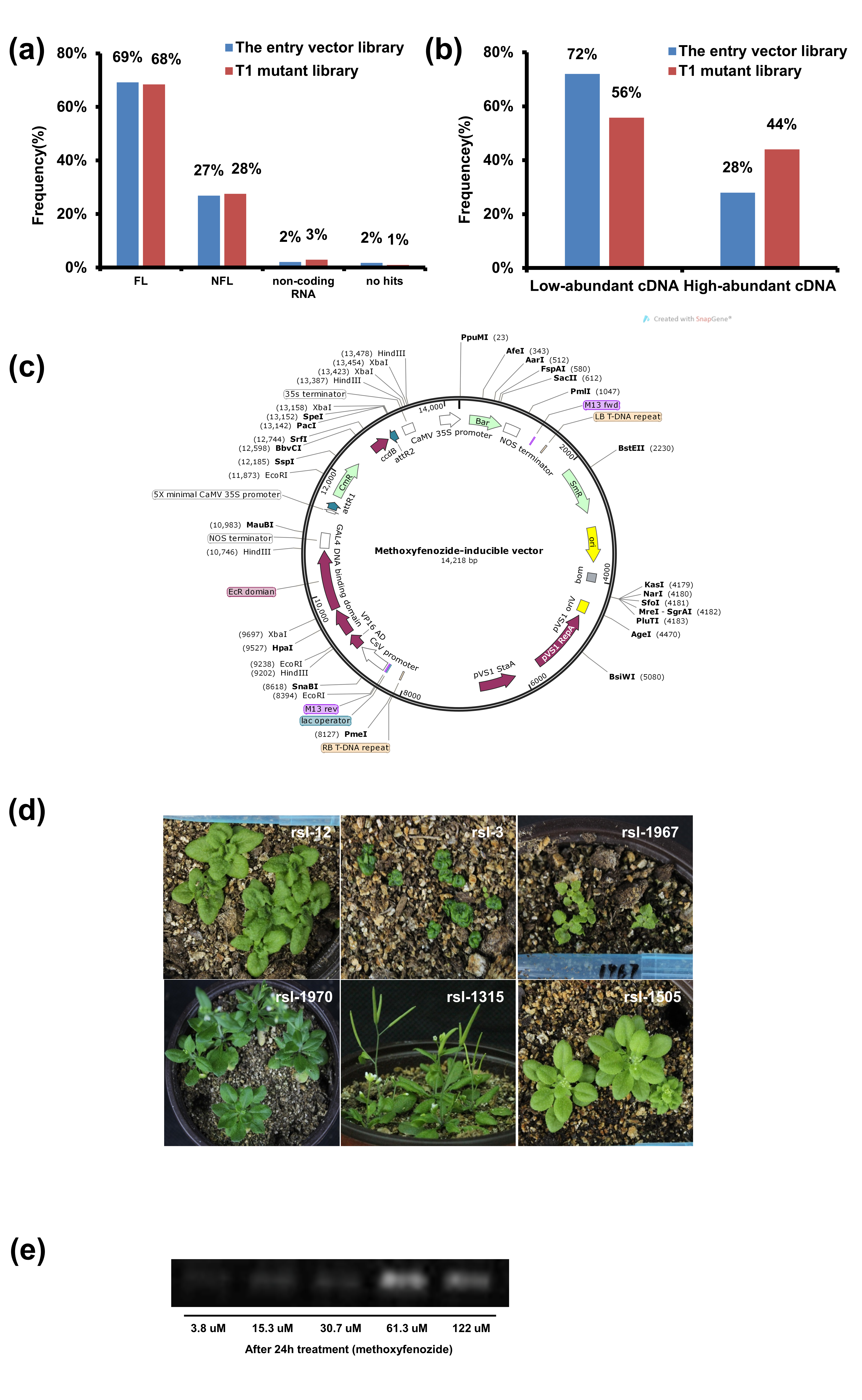

Supplement: Supplementary file 1 — Figure S1 Evaluation of the mutant library and Arabidopsis iFOX line (rsl‐1327). [file PBI-16-591-s016.tif]

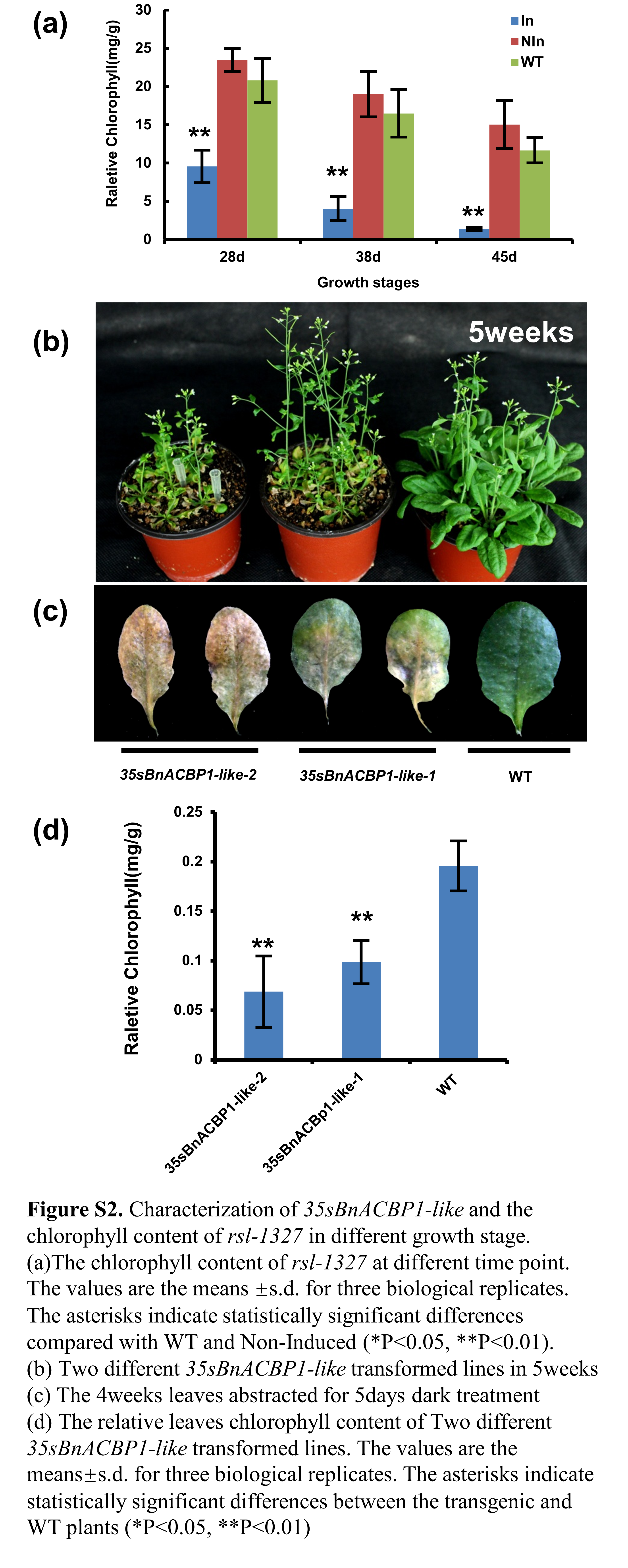

Supplement: Supplementary file 2 — Figure S2 Characterization of 35sBnACBP1‐like and the chlorophyll content of rsl‐1327 in different growth stage. [file PBI-16-591-s015.tif]

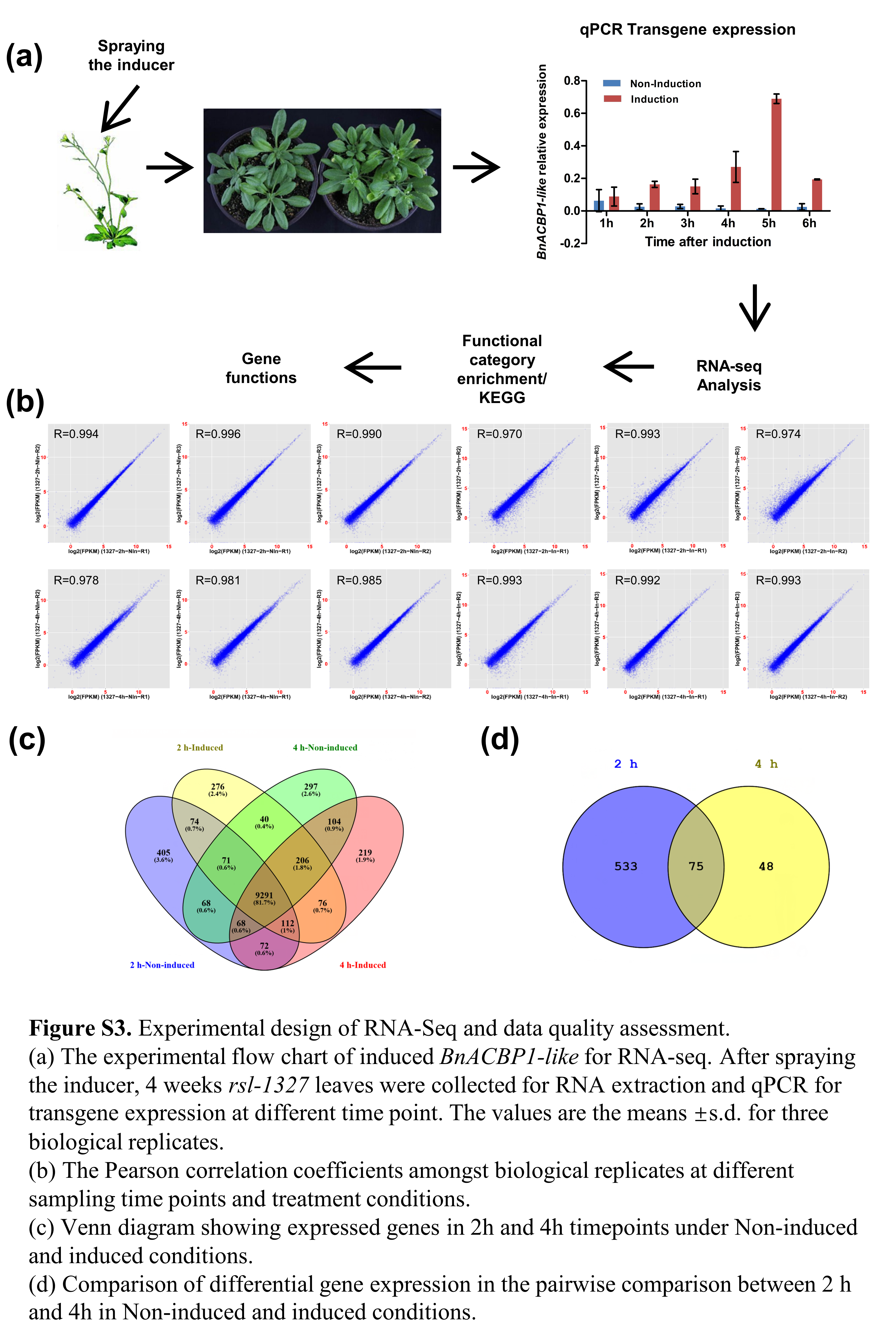

Supplement: Supplementary file 3 — Figure S3 Experimental design of RNA‐Seq and data quality assessment. [file PBI-16-591-s014.tif]

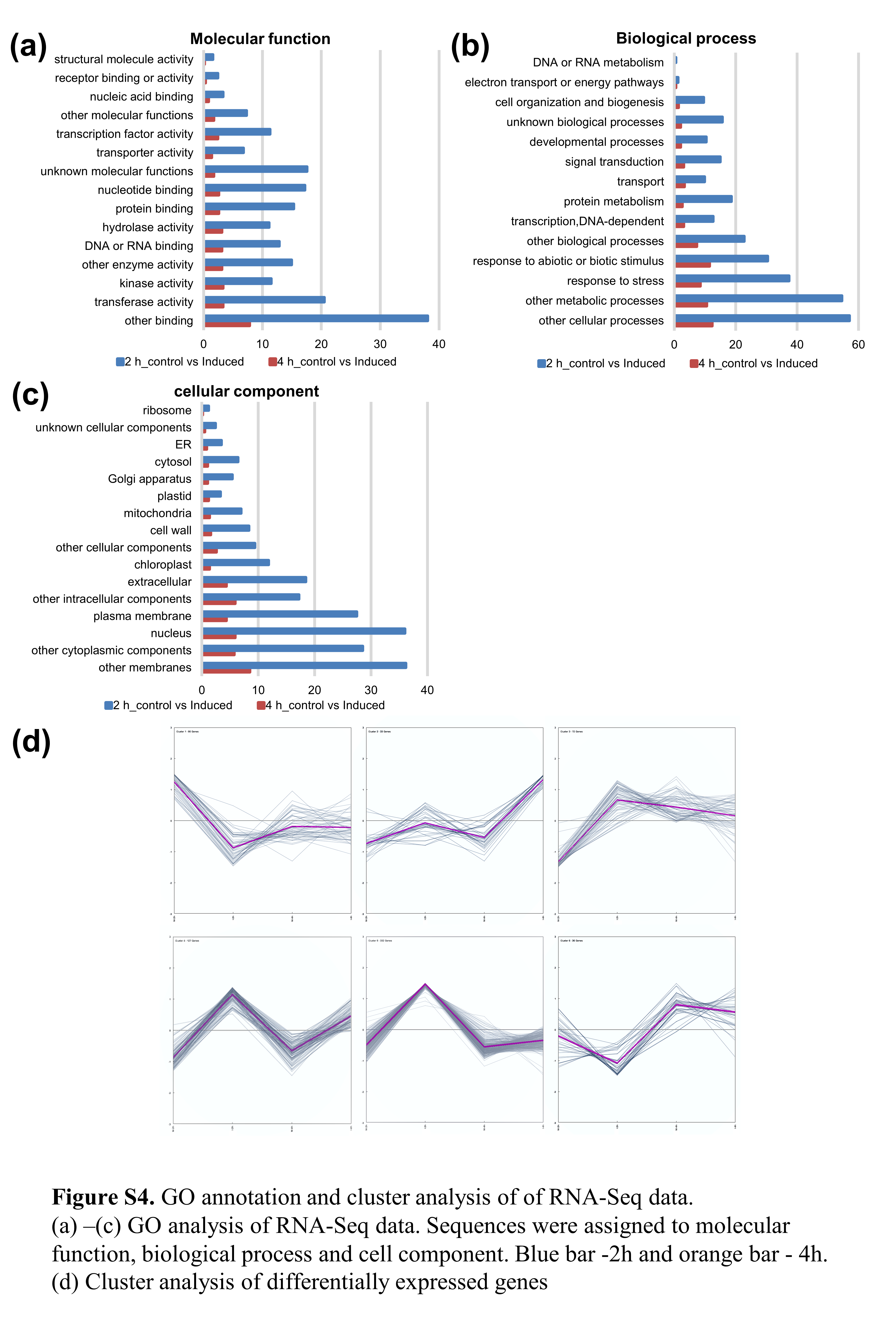

Supplement: Supplementary file 4 — Figure S4 GO annotation and cluster analysis of RNA‐Seq data. [file PBI-16-591-s002.tif]

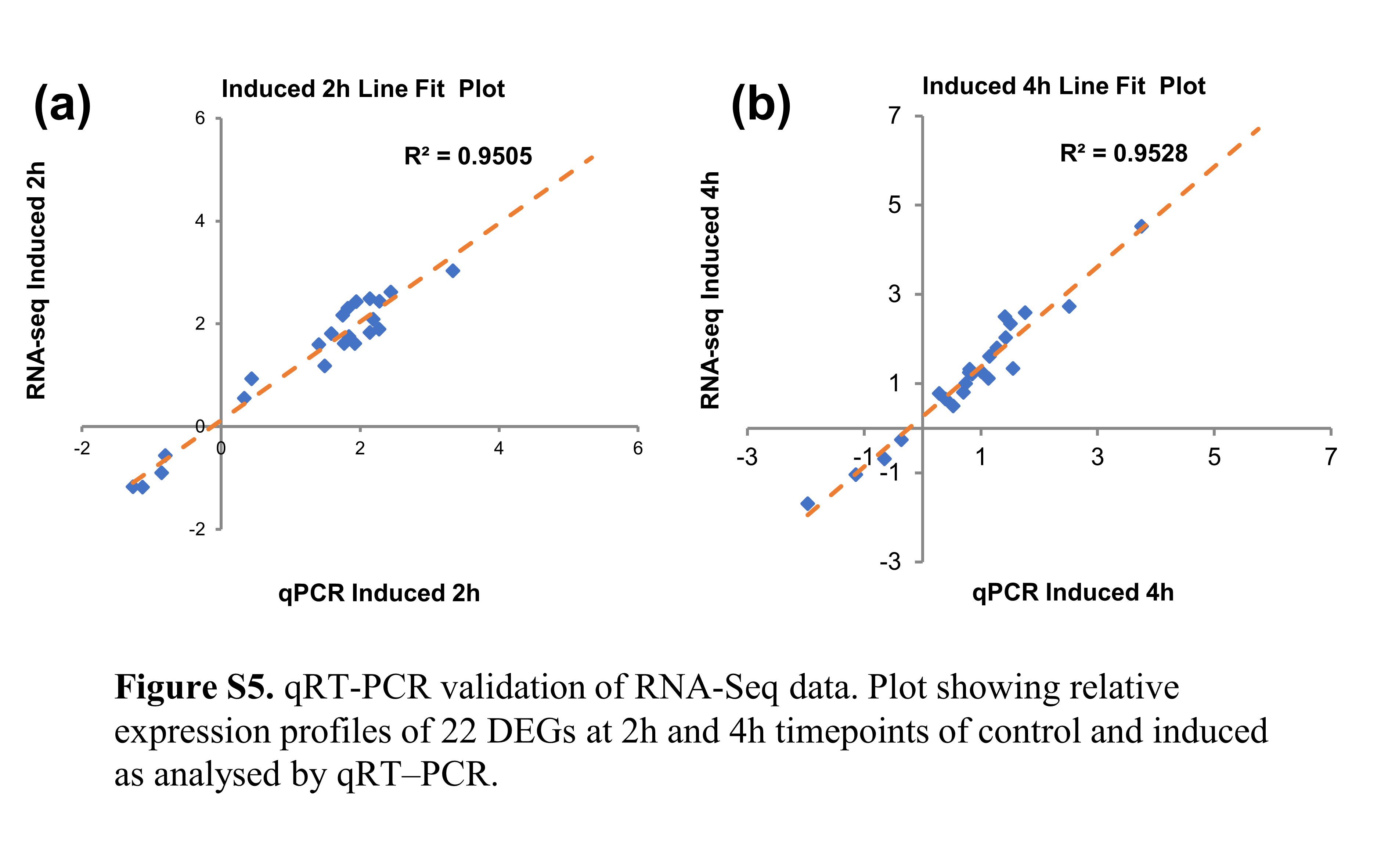

Supplement: Supplementary file 5 — Figure S5 qRT‐PCR validation of RNA‐Seq data. [file PBI-16-591-s001.tif]
