# Supplementary material for: Development of iFOX‐hunting as a functional genomic tool and demonstration of its use to identify early senescence‐related genes in the polyploid Brassica napus
Source: Plant Biotechnol J. 2017 Aug 22;16(2):591–602. doi: 10.1111/pbi.12799 (PMC5787830; doi:10.1111/pbi.12799)
Supplement: Supplementary file 16 — Data S1 Loss of function phenotype and RNA‐Seq data analysis. [file PBI-16-591-s013.docx]

**Supporting result: Loss of function phenotype and RNA-Seq data analysis**

**Loss-of-function phenotype observed in T_2_ iFOX lines**

In this study, we identified 6 loss-of-function mutant lines showing visible phenotypes such as cabbage leaf, shrivelled leaf, leaf malformation and inflorescence differentiation malformation (Figure S1d). We demonstrate that inducibility of integrated rapeseed fl-cDNA in these lines did not confer these phenotypes compared to gain-of-function mutant phenotypes, rather the loss-of-function phenotype resulted from T-DNA insertion. As shown in Figure S1d, the same phenotype is maintained both in induced and non-induced state as. This is another powerful advantage of our iFOX system, i.e. it is easy to distinguish phenotypes that arise from T-DNA insertion than induction of fl-cDNA. Nevertheless, these loss-of-function lines could be useful resources for rapeseed functional genomics study.

**RNA-Seq data analysis**

The result of quality assessment of the sequenced data is reported in Table S4. The Pearson correlation coefficients (R > 0.98; Figure S3b) amongst biological replicates at different sampling time points and treatment conditions (Non-induced and Induced), demonstrate the high quality of our RNA-Seq data. Mapping results indicates a very high concordant pair alignment rate (>91%) Table S4.

For gene expression analysis, an arbitrary lower limit of detection for expression estimate was designated to be an FPKM of 1 or if the FPKM value was less than 1, at least 50 uniquely mapped reads with identity >98% over 100 bp. Overall, our data-set identified approximately 86% of Arabidopsis annotated transcripts (representing 23136 genes) expressed throughout the time course under study, and about 82% of all the expressed genes was shared in both treatment conditions (Figure S3c). Additionally, about 2.4% (representing 276 genes) and 1.9% (representing 219 genes) were exclusively expressed in the Induced treatment condition at 2 and 4 hr, respectively. Similarly, 3.6% (representing 405 genes) and 2.6% (representing 297 genes) were uniquely specific in the control treatment at 2 and 4 h, respectively (Figure S3c).

**Functional classification and cluster analysis of differentially expressed genes (DEGs)**

We used gene ontology (GO) annotations of the TAIR database for GO analysis of RNA-Seq data. DEGs in both treatment condition and time points were searched and sequences were assigned to molecular function, biological process and cell component (Figure S4a-c). To highlight important differential expression pattern among DEGs, we divided all 656 DEGs from the two time points and treatment conditions into 6 clusters using Genesis software programme based on the K-means clustering method. The results revealed significant co-expression relationship among DEGs in early response to the induction of *BnACBP1-like* (Figure S4d). Cluster 1 had genes that were significantly repressed at 2 h after inducer treatment but had no significant changes afterwards. Interestingly cluster 2 contained genes with reduced expression level at 2 h after induction but were highly upregulated 4 h after induction. Cluster 3 is made up of significantly upregulated genes 2 h after treatment that maintained a stable expression afterwards. Clusters 4 and 5 consisted of specifically positively regulated genes along the whole-time course. Clusters 6 had genes that were significantly repressed at 2 h after treatment but were significantly upregulated 4 h after inducer treatment.

**Validation of DEGs by quantitative real-time reverse transcription–PCR (qRT–PCR)**

To validate the results obtained by RNA-Seq, relative expression profiles of 22 genes were analysed by real-time PCR in the control and induced (Figure S5a and b). The results obtained from qPCR experiment highly correlated with RNA-Seq for both treatment conditions and sampling time points (R = 0.881–0.990, Figure S5a and b), with biological replicated showing consistent expression profile. This demonstrating the reliability of our RNA-seq results.
